# Data standards for single‐cell RNA‐sequencing of paediatric cancer

**DOI:** 10.1002/cti2.70033

**Published:** 2025-05-23

**Authors:** Xiaohan Xu, John Saxon, Megan Sioe Fei Soon, Colin YC Lee, Zewen Kelvin Tuong

**Affiliations:** ^1^ Ian Frazer Centre for Children's Immunotherapy Research, Child Health Research Centre, Faculty of Health, Medicine and Behavioural Sciences The University of Queensland Brisbane QLD Australia; ^2^ School of Clinical Medicine University of Cambridge Cambridge UK

**Keywords:** community, paediatric cancer, repository, RNA‐sequencing data, single‐cell

## Abstract

Single‐cell RNA sequencing (scRNA‐seq) is a powerful tool for investigating paediatric cancers, but individual studies often profile a small number of individuals. It is now the standard practice to upload the scRNA‐seq data to data repositories to support scientific reproducibility. Public data deposition is a cost‐effective and sustainability‐conscious solution that allows any researcher to download and analyse existing scRNA‐seq data to develop new ideas. This is incredibly valuable, especially in the context of paediatric cancer research, where access to funding and to patient cohorts may be prohibitive. However, standards for data deposition are absent, leading to significant issues that may slow progress. As a consequence, it is difficult, even impossible, for other researchers to validate findings or utilise these data for tailored analyses. Here, we systematically accessed and reviewed publicly available scRNA‐seq data sets from various paediatric cancer studies, covering over 1.3 million cells across 488 clinical samples. We highlight striking inconsistencies with study design and data availability across several levels, which hinder downstream analyses and data reproducibility. To address these challenges, we propose a recommendations framework to improve data deposition practices that promote more effective use of scRNA‐seq data sets deposited on public repositories and accelerate discoveries in paediatric cancer research and beyond. We urge data standards institutes and repositories, such as NCBI Gene Expression Omnibus (GEO) and European Genome‐Phenome Archive (EGA), to strictly enforce these standardised data practices.

## Graphical abstract

Publicly available scRNA‐seq data can support new research ideas, especially in resource‐limited settings, but the lack of standards complicates data validation and analysis. In this article, we review scRNA‐seq data sets from paediatric cancer studies, identifying issues with study design and data availability that impede reproducibility. We propose a framework to improve data deposition practices, aiming to enhance the use and impact of publicly available scRNA‐seq data in paediatric cancer research.

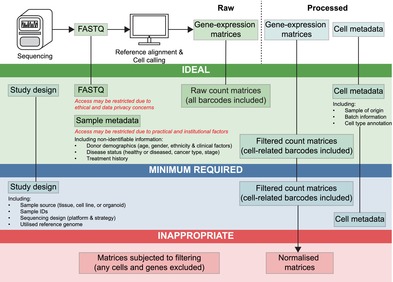

## Introduction

Paediatric cancers are a leading cause of mortality in children and adolescents, with approximately 400 000 diagnoses annually and a mortality rate of 2.1 per 100 000 children.^1^, ^2^, ^3^ The common cancers include acute lymphocytic leukaemias (ALL), Hodgkin/non‐Hodgkin lymphomas (HL/NHL), central nervous system tumors (CNS, e.g. gliomas and medulloblastoma), bone cancers (e.g. osteosarcoma and Ewing sarcoma), rhabdomyosarcomas, neuroblastomas, retinoblastomas, Wilms tumors and germ cell tumors.^4^ These differ starkly from the cancers that are most prevalent in adults.^5^ Even when the same type of malignancy occurs in both age groups, paediatric cases typically exhibit distinct molecular features. For instance, mutations in *DNMT3A*, *TET2*, *IDH1* and *IDH2* ^6^, ^7^, ^8^ are implicated in adult acute myeloid leukaemia (AML), while paediatric AML is commonly driven by gene changes in *MLL*, *RUNX1*, *CBFB* and *FLT3*.^9^, ^10^ Similarly, adult renal cell carcinomas are most commonly driven by mutations in *VHL*, *PBRM1* and *SETD2* genes in mature renal tubular epithelial cells,^11^, ^12^ whereas Wilms tumors in children are characterised by somatic changes in *WT1*, *CTNNB1* and *WTX* in embryonal kidney cells.^13^, ^14^, ^15^ Overall, these differences have been attributed to the nature of paediatric cancers as developmental diseases. According to the maturation block theory, these cancers are initiated by rare mutations that disrupt normal cell maturation during tissue or organ development.^16^ Further genetic and epigenetic dysregulation promotes uncontrolled proliferation in these immature cells, resulting in tumor formation. In contrast, adult cancers result from the successive accumulation of somatic mutations throughout life that culminates in malignant transformation.^16^ Moreover, paediatric cancers experience reduced exposure to carcinogenic environmental factors, and approximately 90% of paediatric cancers are driven by cryptic somatic mutations that occur during development.^17^ These genetic abnormalities result in highly diverse tumor growth patterns, clinical presentation and treatment outcomes.

Studies on paediatric cancers have also traditionally been challenging because of the absence of standardised criteria or disease definitions. Because of the low frequency of paediatric cancers in comparison with adult cancers, multi‐national cross‐institutional collaborations are often necessary to accumulate sufficient cases for statistical robustness.^18^ However, these endeavours encounter various obstacles including communication barriers, challenges in sharing biological specimens and importantly, inconsistent classification systems adopted across different countries.^18^ Furthermore, many clinical trials are conducted on unstratified children affected by a broad disease phenotype, thereby generating difficulties in interpretation of respective findings and leaving the role of proposed treatments unclear.^19^

**Figure 1:**
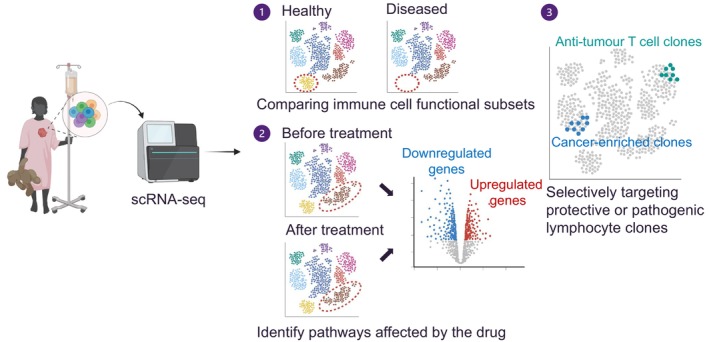
Clinical applications of scRNA‐seq data in paediatric cancer research. scRNA‐seq enables the identification of immune cell subsets critical for disease control (1), reveals gene expression changes before and after treatment to uncover drug mechanisms and resistance pathways (2) and facilitates targeting of specific immune subsets for precise therapeutic strategies (3). This figure was created in BioRender.com.

Recent advances in RNA sequencing (RNA‐seq) technologies have enabled researchers to analyse whole transcriptomes at a single‐cell resolution, known as single‐cell RNA‐seq (scRNA‐seq). A key strength of scRNA‐seq lies in its ability to profile the heterogenous cellular landscape within the tumor microenvironment (TME), thereby conferring broad clinical applications (Figure 1).^20^ Furthermore, scRNA‐seq provides insights into cell states, developmental trajectories during tumourigenesis, cell–cell interactions and potential immune evasion mechanisms of malignant cells, helping to predict treatment responses and inform therapeutic interventions.^21^ This has led to several large collaborative cross‐centre initiatives to profile the cellular landscape of various paediatric cancers, or to create single‐cell ‘atlases’ of the cancers. These included efforts to resolve developmental hierarchy and cellular architecture in paediatric cancers of the central nervous system (CNS) (e.g. gliomas, medulloblastoma and ependymoma)^22^, ^23^, ^24^, ^25^, ^26^ and neuroblastic tumors arising in the peripheral nervous system (PNS, e.g. neuroblastoma).^27^, ^28^, ^29^, ^30^, ^31^ scRNA‐seq has also been harnessed to elucidate the mechanisms underlying treatment outcomes, immune responses and intercellular communication networks in paediatric AML^32^ and bone cancers (e.g. Ewing sarcoma and osteosarcoma).^33^, ^34^, ^35^ However, the high costs of scRNA‐seq and infrequency of paediatric cancers mean that individual single‐centre studies typically analyse a small number of individuals, limiting the statistical robustness of their findings.

## Survey of publicly available single‐cell data of paediatric cancers

The Human Cell Atlas (HCA) project aims to profile gene expression patterns, cellular interactions, developmental trajectories and spatial organisations across the human body's 37 trillion cells. However, only a marginal 2.6% of samples in the HCA are currently derived from children as of 27 November 2024.^36^ This gross underrepresentation underscores the absence of a reliable benchmark or a suitable normative reference for the paediatric biological systems. There have been several initiatives focussing specifically on generating paediatric cell atlases. For instance, the Paediatric Single Cell Cancer Atlas (PedSCAtlas) centres on haematological cancers including AML, ALL and MPAL (mixed‐phenotype acute leukaemia).^37^

As part of federal, funding body and publisher‐driven efforts to promote open science and data reproducibility, it is now standard expectation that RNA‐seq data are deposited on publicly accessible data repositories prior to manuscript publication.^38^ As a result, a vast amount of data are now available to all researchers, with exciting implications for accelerating biological discovery.^39^ Hence, we conducted a systematic literature search using Google Scholar for published scRNA‐seq data sets of paediatric cancers up to March 2024. Search keywords were ‘paediatric cancer’ and specific common paediatric cancer types (leukaemia, lymphoma, brain tumor, bone cancer, rhabdomyosarcoma, neuroblastoma, retinoblastoma, Wilm's tumor and germ cell tumor), combined with ‘single‐cell RNA sequencing’ or ‘scRNA‐seq’. The following inclusion criteria were applied to data sets:

1. The scRNA‐seq data were generated directly from patient biopsies, excluding data from patient‐derived cell lines, organoids, or xenografts.
2. Patients were under 18 years of age at the time of sampling.

For data sets that included both adults and children, only the scRNA‐seq data from paediatric patients were included. Data sets that fulfilled these inclusion criteria are listed in Table 1, including study metadata and the associated paediatric cancer type.

**Table 1:** Basic information of collected scRNA‐seq data sets

	Paper DOI	Accession code	No. of samples (no. of patients)
Leukaemia	10.1038/s41598‐021‐85034‐7	GSE132509	11 (11)
	10.1186/s13073‐023‐01241‐z	GSE236351	7 (7)
	10.1186/s13073‐020‐00799‐2	GSE148218	8 (6)
	10.1038/s41591‐022‐01720‐7	ERP125305 & EGAD00001007854	10 (10)
	10.1038/s41375‐018‐0127‐8	EGAS00001002830	4 (4)
	10.1038/s41556‐021‐00814‐7	HRA000489	24 (10)
	10.1172/jci.insight.140179	GSE154109	15 (15)
	10.1038/s41467‐023‐41994‐0	GSE235923	31 (20)
	10.1016/j.ccell.2023.10.008	GSE235063 & EGAD00001011194	75 (28)
	10.1038/s41598‐023‐39152‐z	GSE227122	16 (11)
CNS tumor	10.1126/science.aao4750	GSE102130	10 (6)
	10.1038/s41586‐019‐1434‐6	GSE119926	25 (25)
	10.1093/neuonc/noab135	GSE155446/GSE156053	30 (28)
	10.1016/j.ccell.2020.06.004	GSE141460	28 (21)
	10.3389/fimmu.2022.903246	GSE189939	4 (4)
	10.1016/j.celrep.2020.108023	GSE125969/GSE126025	26 (26)
	10.1093/neuonc/noad207	GSE231860/GSE231859	19 (19)
	10.1038/s41591‐020‐0844‐1	GSE140819	1 (1)
	10.1038/s43018‐023‐00706‐9	GSE221776	39 (39)
Bone cancer	10.1158/1078‐0432.CCR‐22‐1471	GSE198896	14 (12)
	10.3389/fonc.2021.709210	GSE162454	3 (3)
	10.1038/s41467‐021‐23119‐7	GSE152048	6 (6)
	10.1158/2767‐9764.CRC‐23‐0027	GSE243347	27 (11)
Sarcoma	10.1038/s43018‐022‐00414‐w	GSE195709	4 (4)
	10.1016/j.devcel.2022.04.003	GSE174376	18 (16)
	10.1038/s41591‐020‐0844‐1	GSE140819	4 (2)
	10.1038/s41467‐023‐38886‐8	EGAD00001009385	19 (19)
Peripheral neuroblastic tumor	10.1038/s41588‐021‐00806‐1	EGAS00001004388	31 (31)
	10.1126/sciadv.abd3311	EGAD00001008345 & PRJEB41516 and ERP125307	28 (28)
	10.1111/cas.15707	NA	5 (5)
	10.1016/j.ccell.2020.08.014	GSE137804	22 (22)
	10.1016/j.gendis.2021.12.020	NA	20 (20)
	10.3389/fimmu.2023.1197773	NA	6 (6)
	10.1016/j.celrep.2022.111455	GSE192906	10 (10)
	10.1038/s41591‐020‐0844‐1	GSE140819	8 (4)
	10.1038/s41467‐021‐24870‐7	syn22302605	11 (11)
	10.1136/jitc‐2022‐004807	EGAS00001004781	10 (10)
	10.1038/s41467‐023‐39210‐0	EGAS00001006106 & GSE216176	17 (16)
	10.1016/j.xcrm.2022.100657	GSE147766	19 (17)
	10.1101/2022.07.15.499859	NA	25 (20)
Retinoblastoma	10.1038/s41419‐021‐04390‐4	PRJNA737188	2 (2)
	10.1167/iovs.62.6.18	NA	11 (11)
	10.1038/s41467‐021‐25792‐0	EGAS00001005178	59 (59)
	10.1038/s42003‐023‐05732‐y	GSE249995	4 (4)
	10.1038/s41419‐022‐04904‐8	GSE168434	10 (7)
Kidney cancer	10.1093/ckj/sfad277	GSE223373	3 (1)
	10.1126/science.aat1699	EGAS00001002171, EGAS00001002486, EGAS00001002325, EGAS00001002553	21 (6)
	10.1038/s41467‐021‐23949‐5	EGAD00001004304, EGAD00001007498, EGAD00001007572	NA
Pancreatic cancer	10.1111/cas.15744	HRA002834, PRJCA005331	7 (7)

### Data processing and analysis

**Figure 2:**
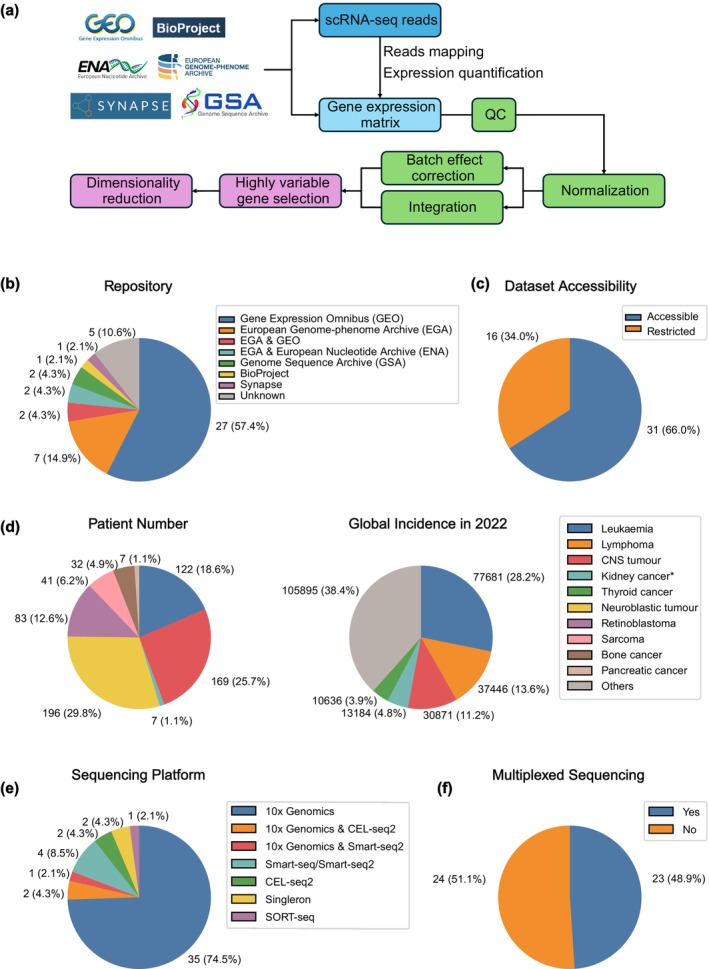
Current publicly available scRNA‐seq data for paediatric cancer research. **(a)** Schematic of study design and processing workflow. **(b)** Pie chart of the distribution of online repositories archiving the identified 47 scRNA‐seq data sets. **(c)** Pie chart of the proportion of publicly accessible data sets. **(d)** Distribution of identified scRNA‐seq data sets for paediatric cancers. The left panel displays the total number of patients recruited per major paediatric cancer type, and the right panel displays the global incidence of paediatric cancers. Data from one study in the kidney cancer cohort, lacking patient numbers, were excluded from the kidney cancer patient count. **(e)** Pie chart of the distribution of scRNA‐seq platforms across 47 identified scRNA‐seq data sets. **(f)** Pie chart of the distribution of scRNA‐seq data sets that incorporated multiplexing or not.

Publicly accessible scRNA‐seq data were downloaded from corresponding online repositories. Analysis was performed using a standardised preprocessing workflow (Figure 2a). For data sets providing raw sequencing data in the format of FASTQ files, CellRanger (v7.2.0) count was used with default settings to align sequencing reads to the human genome reference GRCh38 and quantify gene expression of single cells. For data sets providing post‐alignment data, gene expression matrices were extracted and then manually inspected to exclude matrices that are processed/transformed counts. The resulting raw gene expression matrices underwent a standard Scanpy^40^ (v1.9.8) pre‐processing pipeline on a per‐sample basis. Cells were filtered based on the number of expressed genes (> 200 and < 6000 genes). We implemented a Gaussian Mixture Model (GMM) from the scikit‐learn library (v1.4.1) with two components to classify the cells into two discrete clusters based on the mitochondrial content of the cells and total number of read counts, allowing up to 1000 iterations for convergence. This enabled flexible mitochondrial content thresholding that accounts for sample and cell type variation. Cells that were unbiasedly labelled into the group with higher mitochondrial content and were considered poor quality and removed. Filtered gene expression profiles were normalised to 10 000 counts per cell and log‐transformed following the Scanpy workflow (v1.9.8).

Gene expression matrices of samples from the same paediatric cancer were concatenated to generate a cancer‐specific cell atlas. All cancer‐specific cell atlases were further concatenated to generate a comprehensive pan‐cancer data set containing scRNA‐seq information of various paediatric cancers. Highly variable genes (HVGs) were selected from the pan‐cancer data set accounting for batch effects among samples. The neighbourhood graph was computed using 30 principal components (PCs) and 20 nearest neighbours. UMAP was then applied to visualise the pre‐integrated expression data in a two‐dimensional space, with a minimum distance of 0.3.

### Overview of survey results

Forty‐seven data sets covering seven major cancer types were curated. The scRNA‐seq data sets were sourced from various genomics data repositories (Figure 2b–d), with the Gene Expression Omnibus (GEO) being the most widely used. Some data sets were deposited in repositories with restricted access, for example on the European Genome‐Phenome Archive (EGA) (Figure 2c). We assigned three hierarchical levels to methodically categorise the cancers: broad cancer type by tissue origin (e.g. leukaemia), cancer type (e.g. ALL) and cancer subtype (e.g. B‐cell ALL). Among these, PNS tumors were the most profiled, both in terms of number of data sets and recruited patients (Figure 2d). Notably, lymphoma, a type of haematological cancer that forms solid tumors in lymphoid organs and is the second most common childhood cancer globally,^41^ was relatively under‐studied by scRNA‐seq. This is possibly because surgical resection of affected lymph nodes is not the mainstay of treatment, limiting access to research samples. Additionally, the high curability of paediatric lymphoma, with an estimated 5‐year survival rate exceeding 98%, may have reduced the emphasis on advanced molecular technologies for studying its pathophysiology.^42^ In contrast, CNS and PNS tumors often undergo surgical resection as a first‐line therapy, providing a route for accessing tissue samples for scRNA‐seq.

Droplet‐based 10x Genomics was the most popular scRNA‐seq platform, adopted by more than 74% of studies (Figure 2e), likely because of the high‐throughput capabilities of the technology compared with competing technologies in the years the studies were conducted. To reduce per sample costs in scRNA‐seq via the 10× Genomics platform, many studies have utilised sample multiplexing strategies, for example by including unique oligo‐tagged antibodies and/or by demultiplexing the scRNA‐seq data based on single nucleotide polymorphisms (SNPs) found in individuals^19^, ^26^, ^29^, ^33^, ^34^, ^43^, ^44^, ^45^, ^46^, ^47^, ^48^, ^49^, ^50^, ^51^, ^52^, ^53^, ^54^, ^55^, ^56^, ^57^, ^58^, ^59^ (Figure 2f). More recently, an alternative strategy, split‐pool barcoding, was developed. Here, cells are tagged with a unique combination of oligonucleotide barcodes through iterative rounds of cell splitting and re‐aggregation without the need for physical separation of single cells (e.g. SPLiT‐seq, sci‐RNA‐seq).^60^ This offers significant advantages in both experimental cost and cell throughput. However, none of the collected data sets employed this approach, possibly because of its novelty.

## Inconsistencies in data deposition and challenges in data utilisation

**Figure 3:**
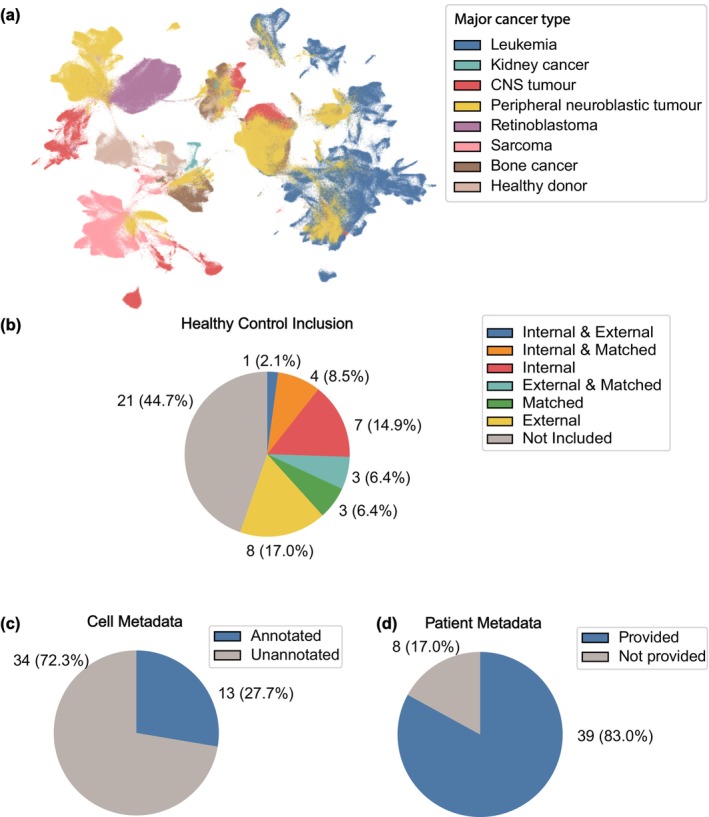
Limitations of sample and metadata availability in scRNA‐seq studies of paediatric cancers. **(a)** UMAP of 1 300 598 cells from 488 samples across 29 studies, covering healthy cells and cells from seven major types of paediatric cancer. Major cancer types are coloured. **(b)** Pie chart of the distribution of data sets including healthy controls for reference. Data sets with internal controls recruited healthy donors in their studies to construct a healthy reference. Data sets with matched controls generated scRNA‐seq from the same patients at different time points, such as before and after treatment, or from different sites, such as tumor samples and proximal healthy tissues. Data sets with external controls used scRNA‐seq data of healthy donors generated by other studies. **(c)** Pie chart of the number of data sets that provide cell annotations for their scRNA‐seq data. **(d)** Pie chart of the number of data sets that provide clinical features of each patient, such as gender and age of sampling.

As previously highlighted, publicly available scRNA‐seq data offer exciting opportunities for research, and democratising access to critical resources such as single‐cell ‘omics’ data promotes data reproducibility and innovation. This is particularly valuable in the field of paediatric cancer, where cases are generally rare, highly heterogeneous in presentation and defined by complex biological/disease traits. However, these opportunities hinge on how the data are shared. Here, we downloaded and processed 29/47 of collected scRNA‐seq data sets that were not subject to restricted access control, generating a data set containing 1 300 958 cells from 488 paediatric cancer samples (Figure 3a). Our attempt at curating these data sets highlighted many important issues hindering productivity, which we discuss briefly below. Unfortunately, some studies did not provide their generated scRNA‐seq data sets in any form.^30^, ^57^, ^61^, ^62^, ^63^

### Lack of controls

First, there is a general lack of inclusion of matched healthy donors as internal reference controls (Figure 3b). It has been shown that when distinguishing altered cell states between disease cohorts and healthy controls, it is more effective to use a combination of data from publicly available healthy single‐cell atlases as well as matched internal controls (i.e. healthy control data generated as part of this study) as the reference than to use either alone.^64^ Lack of appropriate healthy controls may lead to an increase in false‐positive findings of diseased cell states and can misconstrue the biology.^64^ Therefore, researchers that solely analyse disease samples alone are potentially exposed to erroneous discoveries. Integration of publicly available healthy single‐cell atlas data without matched internal control only partially alleviates the issue,^64^ performing worse than with matched controls. This limitation arises because public data sets often introduce batch effects because of differences in cohort characteristics and sequencing protocols. Nonetheless, public data sets are valuable for identifying disease‐relevant genes and distinguishing rare populations. Ideally, a combination of public data sets and matched internal controls provides the most robust reference for disease‐state discovery.

### Feature discrepancies and loss

Second, there are major inconsistencies in the feature space (i.e. genes included in the deposited data) between studies. This issue arises from the deposition of ‘processed’ data by authors, and the term ‘processed’ can be interpreted subjectively. For instance, ‘processed’ data can refer to ‘raw’ integer counts after alignment and gene expression quantification, which could include all recovered barcodes or only those associated with cells identified by the employed cell calling algorithms. While the former gene count matrices provide complete scRNA‐seq results, the latter are still usable for various research purposes. In contrast, some ‘processed’ scRNA‐seq data refer to normalised and/or transformed expression matrices, which, in most cases, cannot be reverted to the ‘raw’ state because of vague descriptions of the preprocessing strategies used in the generation of deposited data sets. In scenarios where a different data normalisation approach is required, these ‘processed’ data sets become unusable. Furthermore, ‘processed’ data may also refer to filtered gene expression matrices that retain only genes expressed in greater than a specified threshold of cells, leading to the loss of information that could be relevant to other researchers. Attempts to integrate these inconsistent data formats can be challenging and often lead to additional feature loss. Feature discrepancies can also arise from the usage of different reference genomes or inconsistent adoption of gene naming conventions. These include the variable use of stable gene identifiers (e.g. Ensembl or Entrez gene identifiers) or gene symbols, modifications made to the feature nomenclature because of multiple identifiers mapping to the same gene symbol, and corruption of gene names because of having processed through popular spreadsheet programs (e.g. Microsoft Excel).^65^ Highlighting the severity of this issue, our pan‐cancer scRNA‐seq object retained only 7662 genes, representing > 77% loss in features (from current standard size of ~33 000 to ~36 000 genes). This reduced feature set likely excludes many biologically relevant gene expression differences and precludes meaningful downstream analysis.

### Lack of important annotations

Another major issue is the absence of essential cell‐level and/or sample‐level metadata availability, accessibility and accuracy. More than 72% of data sets did not provide cell annotations for their scRNA‐seq data (Figure 3c), limiting both the interpretability of the data and the reproducibility of their research findings. Moreover, a striking 83% of data sets did not provide adequate clinical features of each patient sample (e.g. sex, age, disease stage and treatment history) (Figure 3d). It goes without saying that complete clinical metadata is required for the stratification of patient samples to enable meaningful comparisons of disease features between different clinical groups or characteristics. Furthermore, several studies^29^, ^51^ revealed striking inconsistencies in patient identifiers, which posed challenges in accurately correlating clinical background with gene expression profiles. For instance, conflicting information was found between file names storing scRNA‐seq count matrices, supplementary files listing patients' clinical data and sample descriptions in online repositories. In some cases, a single count matrix was linked to multiple patients based on provided sample metadata,^19^ or cells were mapped to unknown samples,^35^ further complicating re‐analysis and limiting the usability of the data.

Overall, researchers will need to improve current practices for metadata deposition to enable the wider scientific community to effectively utilise these data. Data sets that are incomplete, poorly labelled or saved in inconsistent formats create significant barriers to their re‐usability, often requiring extensive efforts to wrangle and reprocess before they can be analysed. We suggest that journals and funders enforce stricter criteria in this regard to promote data transparency, maximise clinical impact and improve sustainability in scientific research. For example, Lambo *et al*.^32^ provided an excellent example by sharing both raw gene expression matrices containing all barcodes and those associated with cells for each donor, along with a metadata file containing cell annotations and relevant patient clinical features. Similarly, Riemonday *et al*.^48^ ensured their data's utility by including a detailed metadata file in the deposited data set, which documents the tumor subgroup and disease progression stage (i.e. primary or recurrence) of each cell, enabling precise patient classification.

## Recommendations to improve scRNA‐seq data usability

Undoubtedly, scRNA‐seq has advanced paediatric cancer research by providing insight into cancer tissues at an unprecedented resolution, and its usefulness is already evident. For instance, Tirosh *et al*.^22^ used scRNA‐seq and identified a rare subpopulation of undifferentiated cells associated with a neural stem cell expression program in paediatric oligodendrogliomas. Similarly, Lambo *et al*.^32^ employed scRNA‐seq to analyse paediatric AML subtypes, generating a highly useful scRNA‐seq resource that spans different clinical timepoints: diagnosis, remission and relapse. Through this data, the authors uncovered that cells emerging during cancer relapse are transcriptionally distinct from malignant cells at diagnosis and that treatment with bortezomib/sorafenib and standard chemotherapy does not fully eliminate multipotent AML blasts.^32^ These are but a few of the many examples that demonstrate the role of single‐cell sequencing in improving our understanding of paediatric cancer biology and its treatment.

Findings from scRNA‐seq have fuelled important discoveries in the field and may lead to clinically actionable interventions. However, just as importantly, these efforts generate invaluable patient‐derived data resources for the paediatric cancer research community, democratising access to precious samples and expensive methodologies. Independent researchers can leverage published scRNA‐seq resources to develop new ideas and test hypotheses, driving further progress in our understanding of these devastating diseases. However, in our review of the currently published scRNA‐seq data for paediatric cancer research, we found a striking lack of organisation and standardisation, including the absence of raw gene expression matrices, incomplete data files, incompatible data formats, inconsistent gene naming conventions and missing cell‐ and sample/patient‐associated metadata. This is despite considerable efforts to generate and maintain consistent and effective data storage and integration solutions by the bioinformatics community, including for gene expression counts and cell/sample‐level metadata, for instance the AnnData format developed by the scverse community^66^ or Seurat^67^ and SingleCellExperiment^68^ formats by R developers. These formats have been adopted by the Human Cell Atlas Data Portal^36^ and by Chan Zuckerberg Initiative,^69^ but overall, deposition of ‘processed’ data is not consistent across other data repositories.

**Figure 4:**
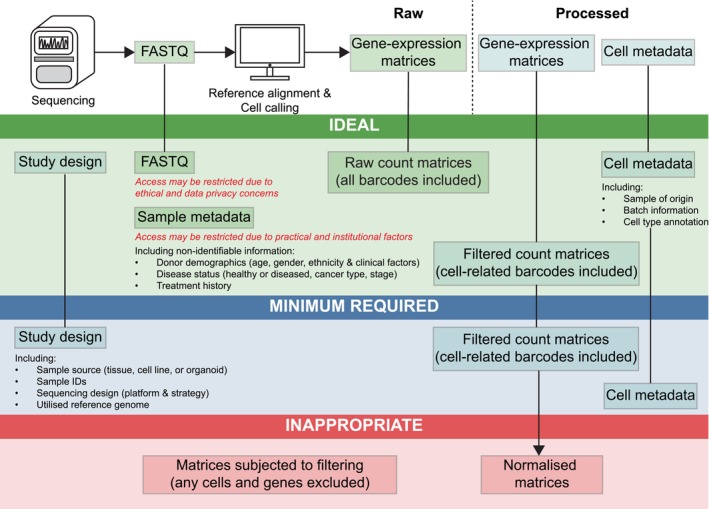
Flowchart for depositing scRNA‐seq data sets in paediatric cancer research. Recommendations to guide researchers on the ideal, minimum required or inappropriate data types for deposition on data repositories. Considering ethical, privacy and administrative factors in data access restriction, ideal files include raw sequencing reads, unfiltered raw count matrices containing all barcodes and all relevant sequencing‐level, cell‐level and sample‐level metadata such as sequencing experiment design, cell‐type annotations and non‐identifiable patient demographics, and original count matrices. At a minimum, raw count matrices containing only cell barcodes should be provided. Post‐processed and/or filtered count matrices are not appropriate and will lead to data loss.

These issues rendered many data sets unusable or necessitated laborious pre‐processing efforts, which may compromise the reproducibility of scientific findings and limit utilisation by the wider scientific community. Furthermore, these issues impede the integration of data sets across studies, limiting statistical power and the discovery of shared molecular mechanisms essential for robust scientific conclusions and clinical advancements. Accordingly, we propose a standardised pathway for depositing scRNA‐seq data sets (Figure 4). We implore data standards institutes and repositories, such as NCBI GEO and EGA, to implement data upload practices, such as the recommendations defined here, to ensure that publicly available data sets are more accessible, reproducible and valuable for future research.

### Upload raw data and associated metadata

As the accuracy and completeness of the human genome reference continue to improve, depositing scRNA‐seq data in the format of raw sequencing reads will future‐proof these data. However, because of ethical concerns and data privacy, raw sequencing reads, which could be patient‐identifiable (e.g. from SNP calling), may be restricted from public access. Consequently, scRNA‐seq data are often deposited as gene expression matrices. During read alignment, cell calling algorithms are used to differentiate between true cell‐associated barcodes and background noise, resulting in both raw and filtered gene expression matrices. Given the ongoing improvements in cell calling algorithms, unfiltered gene expression matrices are preferred. Meanwhile, it is crucial that these matrices contain raw counts as integers rather than normalised, transformed or scaled counts as floats. Processed matrices are not compatible for cross‐study analyses because of the lack of standardisation in processing methods, and they are difficult to revert to their original state. Deposited data should also not have cells and gene/features filtered and/or should be accompanied with code documentation on how the uploaded data were derived, including relevant software versions. However, of note, various practical and institutional factors may affect data access restrictions, such as ongoing clinical trials, where patient confidentiality or blinding is enforced, or data that originate from industry‐led teams. Thus, files containing critical information, including sample IDs, patients' clinical features and cell annotations and uniform manifold approximation and projection (UMAP) coordinates, should be provided as additional meta‐data where possible. Basic non‐sensitive and non‐identifiable data related to the study design should be provided as these are essential even for simple data analyses such as differential gene testing.

## Conclusion

scRNA‐seq and other emerging single‐cell ‘omics’ approaches are uniquely poised to revolutionise precision oncology,^70^ including in paediatric cancer. These technologies offer opportunities to identify biomarkers for disease prognostication and treatment response, tailor therapies to the molecular and cellular features of individual tumors and accelerate the discovery of new therapeutic targets. For most researchers, access to rare clinical samples or single‐cell technologies at scale remains prohibitive, but new regulations on data deposition to promote open science help resolve these limitations.^38^ Indeed, meticulous efforts to deposit complete scRNA‐seq data, including sample‐ and cell‐level documentation,^25^, ^32^, ^48^ have already enabled independent studies to re‐analyse these data to derive novel insight or validate hypotheses. For example, by leveraging over 20 published data sets of various acute leukaemias encompassing over 300 patient samples, Zeng *et al*.^71^ identify conserved patterns in the corruption of bone marrow haematopoiesis during cancer development, underscoring the importance of scRNA‐seq data deposition in increasing sample size and breadth. Moreover, clustering of large disease cohorts using high‐dimensional transcriptomics data enables robust stratification of malignant tissues in a manner that is more sophisticated than standard‐of‐care diagnostic tools used in current clinical practice,^72^, ^73^, ^74^ and scRNA‐seq is now the cornerstone of molecular phenotyping. Certainly, scRNA‐seq will facilitate the movement towards more precise and effective management of paediatric cancers, but promoting consistency in scRNA‐seq data practices will be a critical first step towards its clinical translatability. Finally, despite these opportunities, clinical researchers must be acutely conscious of the threats that RNA‐seq data may pose to patient data privacy and undertake active measures to ensure ethical research standards.^75^

In this review, we undertook a comprehensive survey of 47 scRNA‐seq studies of paediatric cancers and constructed an extensive pan‐cancer atlas from 29 publicly accessible scRNA‐seq data sets covering seven major paediatric cancers. This atlas included gene expression profiles of over 1.3 million cells from 488 clinical samples. Regrettably, substantial challenges were encountered during the curation of these data because of striking inconsistencies in data deposition practices, which reduce their utility. Access to paediatric cancer samples is highly limited, so the re‐analysis of public scRNA‐seq data or integration of published data to increase sample sizes is an important research strategy. To maximise the impact of single‐cell transcriptomics in paediatric cancer research, we recommend a standardised approach to depositing scRNA‐seq data to online repositories. This includes ensuring the completeness of unprocessed gene expression matrices, consistency in data formats, adherence to uniform gene naming conventions and the provision of comprehensive cell and patient metadata. While this review focused on paediatric cancers, the issues we highlight are probably generalisable to all single‐cell research. Adopting better practices in facilitating data availability will be crucial in enabling the broader research community to draw more statistically robust conclusions and drive future discoveries in paediatric oncology.

## Author contributions

**Xiaohan Xu:** Data curation; formal analysis; writing – original draft; writing – review and editing. **John Saxon:** Data curation. **Megan Sioe Fei Soon:** Supervision; writing – review and editing. **Colin YC Lee:** Writing – review and editing. **Zewen Kelvin Tuong:** Conceptualization; project administration; supervision; writing – review and editing.

## Conflict of interest

All authors do not have conflicts of interest to declare.

## Data Availability

The Jupyter Notebooks for extracting gene expression matrices from accessible data sets and preprocessing and meta‐analysis of constructed pan‐cancer scRNA‐seq object are available on GitHub at https://github.com/tuonglab/pan‐paedcancer‐scrnaseq. These data were derived from the resources available in the public domain, as listed in Table 1.
