# Data standards for single‐cell RNA‐sequencing of paediatric cancer

**DOI:** 10.1002/cti2.70044

**Published:** 2025-07-10

Xiaohan Xu, John Saxon, Megan Sioe Fei Soon, Colin YC Lee & Zewen Kelvin Tuong

**Correction to:** Clin Transl Immunol 2025; 14: e70033. https://doi.org/10.1002/cti2.70033

There is an error within a sentence within the first paragraph of the ‘Lack of important annotations’ section, as follows:

Moreover, a striking 83% of data sets did not provide adequate clinical features of each patient sample (e.g. sex, age, disease stage and treatment history) (Figure **3d**).

This should have read:

Moreover, 17% of data sets did not provide adequate clinical features of each patient sample (e.g. sex, age, disease stage and treatment history) (Figure **3d**).

The authors apologise for this error.

